# Pathologic femoral fracture due to tenofovir-induced Fanconi syndrome in patient with chronic hepatitis B: A case report

**DOI:** 10.1097/MD.0000000000008760

**Published:** 2017-11-17

**Authors:** You-Sung Suh, Dong-il Chun, Sung-Woo Choi, Hwan-woong Lee, Jae-Hwi Nho, Soon-Hyo Kwon, Jae-ho Cho, Sung Hun Won

**Affiliations:** a Center of Arthroplasty; b Department of Orthopaedic Surgery; c Department of Nephrology, Soonchunhyang University Hospital Seoul, Seoul; d Department of Orthopaedic Surgery, Chuncheon Sacred Heart Hospital, Hallym University, Chuncheon-si, Gangwon-do, Korea.

**Keywords:** chronic, Fanconi syndrome, femoral fractures, fractures, hepatitis B, spontaneous, tenofovir

## Abstract

**Rationale:**

We report a case of a hepatitis B virus (HBV)-positive patient with preexisting bone disease who developed tenofovir-induced Fanconi syndrome and subsequently sustained pathologic fracture. To our best knowledge, this is the first report in the English literature about pathologic femoral fracture due to tenofovir-induced Fanconi syndrome in patient with chronic hepatitis B (CHB). The present report describes detailed our experience with the diagnosis of pathologic femoral fracture due to tenofovir-induced Fanconi syndrome and treatment.

**Patient concerns:**

A 45-year-old man visited our hospital with pain in the right thigh region and gait disturbance which had started 3 months ago and worsened 1 week before admission. The patient was diagnosed with CHB in 2004. He was on lamivudine medication for 2 years. Medication for the patient was subsequently changed to adefovir in 2009 and tenofovir disoproxil fumarate (TDF) in 2013. He was on TDF since 2013.

**Diagnosis:**

His hip joint magnetic resonance imaging (MRI) revealed hypointensity lesions and cortical bone destruction in fat-saturated MR image at the iliopsoas muscle attachment site of the lesser trochanter of both femur. On blood test showed 25-OH vitamin D level at 6.42 ng/mL (normal range, >20 ng/mL) and U-deoxypyridinoline level at 7.60 nM/mMcr (normal range, 2.30–5.40 nM mMcr). However, osteocalcin and parathyroid hormone levels were within normal range. Based on these findings, the present case was concluded as tenoforvir-induced Fanconi syndrome.

**Interventions:**

TDF treatment was discontinued. After cooperation with internal medicine department, in order to prevent further fractures of the right lesser trochanter, internal fixation was performed under spinal anesthesia using compression hip nails (APIS, TDM, Korea).

**Outcomes:**

Positive outcome by medication and operation demonstrates that his phosphorus and serum calcium levels were maintained within normal range and pain in the right thigh region was improved from visual analogue pain score (VAS) 7 before surgery to VAS 2 after surgery.

**Lessons:**

Physicians need to regularly monitor bone metabolism in patients with take in tenofovir for early diagnosis before its progression to pathologic fractures.

## 1 Introduction

Tenofovir disoproxil fumarate (TDF) is widely used in the world as an effective 1st-line treatment option for chronic hepatitis B (CHB).^[1]^ Although TDF shows excellent efficacy in viral suppression, its long-term use is associated with renal dysfunction.^[2]^ Fanconi syndrome is one of renal dysfunctions in patients receiving TDF treatment. Fanconi syndrome is characterized by proximal tubular dysfunction, leading to increased urinary excretions of phosphorus, glucose, and bicarbonate. Its clinical manifestation includes bone loss, osteomalacia, and electrolyte imbalance.^[2]^

Although uncommon, nephrotoxicity has been reported in about 1% to 2% of human immunodeficiency virus infected patients receiving TDF treatment.^[3]^ Fanconi syndrome in TDF-treated patients with hepatitis B virus (HBV) monoinfection is also reported recently.^[4]^ However, there has been no report of Fanconi syndrome associated with preexisting bone disease leading to pathologic fracture in TDF-treated patient with HBV monoinfection. Here, we present a case of femoral fracture due to TDF-induced Fanconi syndrome in TDF-treated patient with HBV monoinfection.

## 2 Case description

This case report was approved by the Institutional Review Board of Soonchunhyang University Hospital.

A 45-year-old man visited our hospital with pain in the right thigh region and gait disturbance which had started 3 months ago and worsened 1 week before admission. The patient was diagnosed with CHB in 2004. He was on lamivudine medication for 2 years. Medication for the patient was subsequently changed to adefovir in 2009 and TDF in 2013. He was on TDF since 2013. The patient was diagnosed with diabetes 8 years ago. Since then, he has been taken antidiabetic agents. He had no history of hypertension or tuberculosis. He had no history of other hospitalization, surgery, or trauma. He had a drinking history (one and half bottle of “soju” once a week). He was a nonsmoker. He did not take other drugs such as analgesics or herbal medicines. He was not on healthy foods or folk remedies.

In a systematic medical examination, he complained of myalgia in the right thigh region and gait disturbance. He also complained of mild pain in the left thigh region. His vital signs at admission were stable. Physical examination showed that the abdomen was soft without stiffness. There was no hepatosplenomegaly or jaundice. There was no edema in bilateral lower extremity. No limited range of motion of joints or paresthesia was observed. Blood test at admission showed hemoglobin level of 13.3 g/dL, hematocrit of 41.0%, leucocyte count of 8600/μL, platelet count of 183,000/μL, serum sodium level of 137 mmol/L, serum potassium level of 3.7 mmol/L, aspartate aminotransferase level 14 U/L, alanine aminotransferase level of 20 U/L, and serum total bilirubin level of 0.5 mg/dL, all of which were within normal range. He had an increased alkaline phosphatase level at 288 U/L. He was HBsAg-positive. His HBV DNA titer was less than 20 IU/mL.

His blood urea nitrogen and glomerular filtration were within normal range (at 19.9 mg/dL and 68.43 mL/min, respectively). However, his serum creatinine level was increased (at 1.26 mg/dL). Therefore, his previous serum creatinine levels were checked. His serum creatinine level at the time when his medication was changed to adefovir was within normal range (at 1.0 mg/dL). However, his creatinine levels had steadily increased (between 1.2 and 1.5 mg/dL) after taking TDF. His serum calcium level was within normal range (at 9.0 mg/dL). His phosphorus and uric acid levels were at 2.0 and 1.4 mg/dL, respectively, indicating hypophosphatemia and hypouricemia. Urinalysis revealed urine glucose 3+ and trace proteinuria. Urine chemistry test showed that Na level of 85 mmol/L and creatinine level of 112.3 mg/dL. A 24-hour urinalysis showed that proteinuria level was 0.874 g/day and 24-hour urine phosphorus level was 881.0 mg/day. Fractional excretion of potassium was slightly increased by 17.7%.

**Figure 1:**
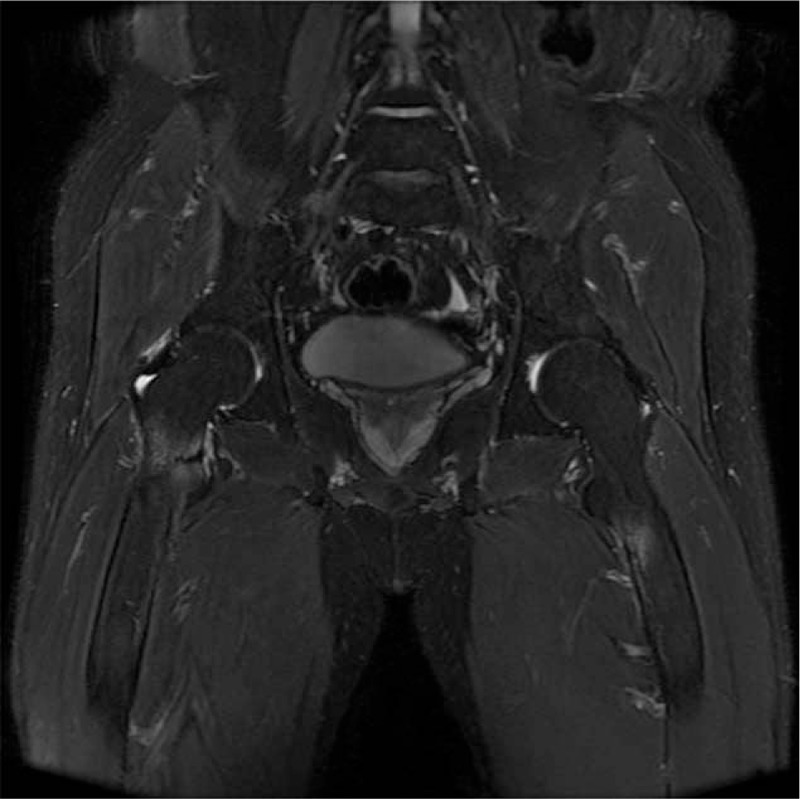
Fat-saturated coronal magnetic resonance (MR) image revealed hypointensity lesions and cortical bone destruction at the iliopsoas muscle attachment site of the lesser trochanter of right femur. Similar lesion can observe in the lesser trochanter of left femur.

**Figure 2:**
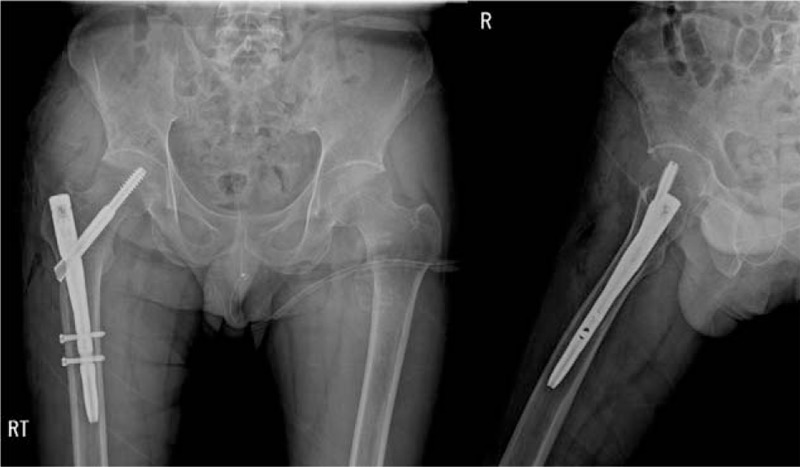
In order to prevent further fractures of the right lesser trochanter, internal fixation was performed using compression hip nail.

Pelvis AP and both hip joint lateral radiographs showed no specific finding. Hip joint MRI revealed hypointensity lesions and cortical bone destruction in fat-saturated MR image at the iliopsoas muscle attachment site of the lesser trochanter of right femur. Similar lesions were observed in the lesser trochanter of left femur. However, no cortical bone destruction was observed (Fig. 1). Bone mineral density test showed that T-scores of L1–2 and femur trochanter were −3.2 and −5.7, respectively, indicating severe osteoporosis. In order to prevent further fractures of the right lesser trochanter, internal fixation was performed under spinal anesthesia using compression hip nails (APIS, TDM, Korea) (Fig. 2).

**Figure 3:**
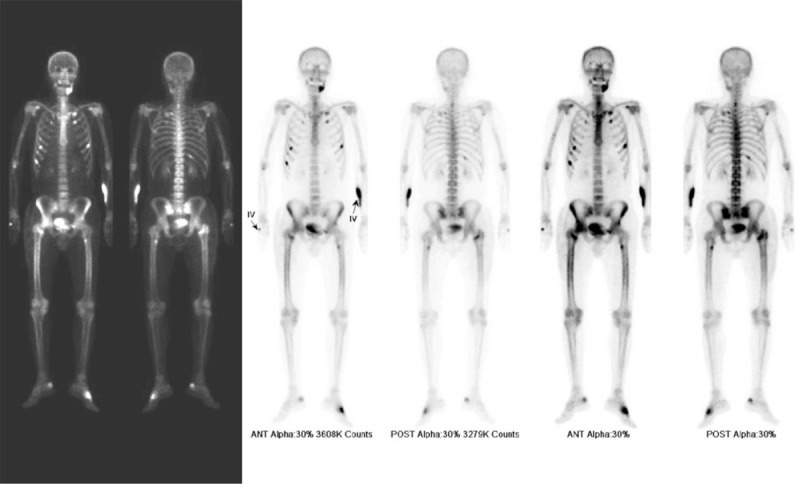
Osteomalacia was observed in the bilateral femoral lesser trochanter, left superior ramus, bilateral sacrum ala, bilateral ribs on bone scan, and SPECT. SPECT = single-photon emission computed tomography.

On bone scan and single-photon emission computed tomography to differentiate severe osteoporosis and nonspecific fractures, osteomalacia was observed in the bilateral femoral lesser trochanter, the left superior ramus, the bilateral sacrum ala, and bilateral ribs (Fig. 3). Blood test showed 25-OH vitamin D level at 6.42 ng/mL (normal range, >20 ng/mL) and U-deoxypyridinoline level at 7.60 nM/mMcr (normal range, 2.30–5.40 nM/mMcr). However, osteocalcin and parathyroid hormone levels were within normal range (at 22.23 ng/mL and 54.24 pg/mL, respectively).

Based on these findings, the present case was concluded as tenoforvir-induced Fanconi syndrome. Therefore, TDF treatment was discontinued. After cooperation with internal medicine department, high phosphorus and high calcium diets were performed. Oral phosphorus and calcitriol were concurrently administered. After 2 weeks, his serum creatinine remained high at 1.32 mg/dL. However, his phosphorus and serum calcium levels were maintained within normal range (at 2.6 and 9.5 mg/dL, respectively). Pain in the right thigh region was improved from visual analogue pain score (VAS) 7 before surgery to VAS 2 after surgery.

## 3 Discussion

High intracellular concentrations of TDF are known to interact with mitochondrial DNA.^[5]^ Thus, TDF is an effective 1st-line agent for patients with CHB who are resistant to lamivudine treatment.^[1]^ However, long-term use of TDF is known to cause renal dysfunction. The cause of renal dysfunction in patients treated with TDF is known to be secondary to mitochondrial DNA toxicity within proximal tubules.^[6]^ Dysfunction of the proximal renal tubules due to TDF treatment can lead to urinary excretion of phosphorus and inhibit 1a-hydroxylation of vitamin D in the kidney.^[7]^ The inhibition of 1a-hydroxylase in the kidney will then inhibit the synthesis of 1.25(OH)_2_ vitamin D (calcitriol).^[8]^ Therefore, clinical findings of osteoporosis in this patient are thought to be secondary osteoporosis due to osteomalacia caused by TDF treatment. Such osteomalacia has been rarely observed in previous case reports of Fanconi syndrome due to TDF treatment in patients with HBV infection.^[4]^ Moreover, reports of pathologic fractures due to severe osteoporosis at a relatively young age as in this case are extremely scarce.

In order to avoid renal toxicity in HBV infected patients undergoing TDF treatment, physicians need to identify coexisting risk factors for renal toxicity and regularly monitor serum creatinine and phosphorus levels. Dose adjustment according to creatinine clearance (CrCl) is also important. The American Association for the Study of Liver recommends that patients receiving TDF treatment should be monitored for their serum creatinine, serum phosphorus, urine glucose, and urine protein levels at least once a year.^[9]^ More frequently monitoring should be performed for those with risk factors for renal dysfunction. The European Society for the Study of the Liver recommends monitoring serum creatinine and phosphorus level every 3 months in the 1st year of TDF treatment and every 6 months thereafter. It recommends monitoring those with high renal risk every month in the 1st 3 months of TDF treatment and every 3 months up to 1 year after that. Then they should be followed up every 6 months thereafter.^[1]^

TDF treatment is known to increase bone turnover but decrease bone mineral density.^[10]^ A study by Gallant et al^[10]^ have reported that TDF is significantly associated with bone loss compared to other antiretroviral agents. However, bone loss is not a progressive phenomenon. It is so small that it is not obvious clinically. Although TDF is known to be associated with osteopenia, reports of bone disease in TDF-treated patients with chronic HBV monoinfection are rare. Furthermore, there has been no report of pathologic fractures in TDF-treated patients as found in the patient in the present case report. The present case report will raise awareness of bone disease in patients with CHB when physicians prescribe TDF.

## 4 Conclusion

Physicians need to regularly monitor renal toxicity and bone metabolism in patients with take in tenofovir for early diagnosis before its progression to pathologic fractures.

## Acknowledgments

The authors thank Soonchunhyang University Research Fund for the support.
